# In Evaluating Technological Risks, When and Why Should We Consult Our Emotions?

**DOI:** 10.1007/s11948-020-00194-5

**Published:** 2020-02-11

**Authors:** Sven Nyholm

**Affiliations:** Ethics Institute, Utrecht University, Utrecht, The Netherlands

In December of 2018, the *New York Times* ran a story about members of the public in Arizona attacking experimental self-driving cars that were being road-tested on public streets there (Romero 2018). Some people were reportedly throwing rocks at the cars. Others were slashing their tires, or waving guns at them. Why the anger at these self-driving cars (or at the companies experimenting with them on public roads)? Some Arizonans felt put at risk. In March of the year before, an Arizona native was hit and killed by an experimental self-driving car operated by the company Uber. This clearly illustrated the complications of testing out these new technologies among ordinary people, who incidentally had not consented to participating in this experiment. One Arizona man, Mr. O’Polka, was quoted as saying the following in the article, “They said they need real-world examples, but I don’t want to be their real-world mistake.” These Arizonans responded to these technological risks with anger and fear. They apparently felt they were being wronged or treated unfairly. What, if anything, could these Arizonans learn about the ethical dimensions of their situation by consulting their emotions about the risks they were being exposed to?

Sabine Roeser’s rich and stimulating book *Risk, Technology, and Moral Emotions* is about exactly this sort of question (Roeser 2018). On a general level, the book is about three main topics: (1) the ethical assessment of technological risks, (2) the role of emotions in such risk assessments, and (3) what meta-ethical and moral–psychological theories best make sense of the role(s) that emotions do and should play in technological risk assessments. On the level of first-order normative ethics, Roeser opposes narrow, monistic views about what considerations should matter in risk assessments, arguing instead for a broad, pluralistic view. She argues that when we allow our emotions to guide us in our assessments of technological risks, this makes us shrink from narrow, technocratic risk-assessments. Risk assessment should not only be about utilitarian cost–benefit analysis. It should instead take into account a richer range of ethical considerations, like fairness, autonomy, equality, and the like. Roeser argues that if and when we consult our own emotions about risky technologies, most of us will agree with her.

In what follows, I will focus on Roeser’s meta-ethical defense of consulting our emotions in ethical assessments of technological risks. I will identify two different parts of Roeser’s defense of the role of emotions: firstly, that emotions “point” us towards our values and, secondly, that emotions are “perceptions of […] moral aspects of the world” (Roeser 2018, 2; 40). Of these two, I will argue that only the first part is needed to achieve Roeser’s normative aims. This is a good thing. The second part of Roeser’s defense of the role of emotions in ethical risk assessment is highly controversial. Most philosophers would reject that second part. If it were needed in order for a defense of consulting our emotions in risk assessments to succeed, Roeser’s key normative claims would rest on much shakier grounds than they actually do. Luckily, as things stand, the more controversial parts of Roeser’s views are optional. We can disagree with them even as we agree with Roeser that the ethics of risk assessment should take into account a wide range of considerations and do so in a way that is partly guided by our emotions.

I start below by explaining what kinds of views Roeser is arguing against, so as to provide more context for her own view (“Views Roeser Argues Against” section). I then say more about what Roeser says on behalf of emotions as a source of moral insight in the ethical evaluation of technological risks. In doing so, I separate out the two different parts of Roeser’s defense quickly mentioned above, and I explain why I think the first part is sufficient for Roeser’s normative purposes (“Roeser’s Defense of Risk Assessment Based on Emotions” section). I then turn to the second, more controversial part of Roeser’s defense. I contrast it with some other meta-ethical views, and argue that this part is not needed for Roeser’s overall defense of the role of emotions in risk assessment to succeed (“The Controversial Nature of Roeser’s Meta-ethics” section). Lastly, I consider one more aspect of Roeser’s own motivation for that part of her view, and argue against it (“A Possible Reply from Roeser” section).

## Views Roeser Argues Against

I have already mentioned that on the level of first-order normative ethics, Roeser argues against narrow views that only focus on small ranges of ethical considerations (e.g. lives saved vs. lives lost), and thereby exclude important values like fairness, autonomy, and equality. Roeser also argues against various other views, on different levels of abstraction.

For example, those whom she calls *technocrats* ignore emotions and many common sense values. They focus exclusively on formal, quantitative approaches to risk assessment, relying heavily on expert judgments about risks, while excluding ordinary people’s opinions. Roeser objects that such views are questionable both from the point of view of democratic legitimacy and from the point of view of several core values typically appealed to within ordinary ethical reasoning, such as fairness, autonomy, and equality (Roeser 2018, 14–18).

Those whom Roeser calls *populists* agree with technocrats that people’s emotions are irrational, but nevertheless think that ordinary people and their emotions should be paid attention to in risk assessments. The idea here is that public support should always be sought when decisions are made. But since the public is largely irrational, what is needed is (what might be called) brute public support, rather than any extensive public debates. In Roeser’s judgment, such views also fall short of the ideal of democracy, which should involve public debates and reason-giving (Roeser 2018, 18–21).

Defenders of what Roeser calls *participatory approaches*, in turn, agree that emotions are subjective or irrational. But at the same time, they are also relativists or subjectivists about the sorts of facts and theories that scientists and other experts operate with. On such views, members of the general public and experts are seen as equally “biased” in their judgments. Therefore, everyone’s opinions should be heard and included in assessments of technological risks. This type of view is doubly offensive to Roeser, who herself both respects experts and scientists, on the one hand, and members of the public, on the other hand. Moreover, values are represented as arbitrary on these views—a stance that Roeser certainly does not agree with (Roeser 2018, 21–22).

Roeser also takes issue with social scientists who assume that values are subjective or socially constructed (Roeser 2018, 37). They have no right to simply assume this in their work, as Roeser understands many of them as doing. The view that values are subjective or socially constructed is a philosophical view needing a philosophical defense, not something that can just be taken for granted. Roeser appears to be particularly frustrated with the influential risk researcher and social scientist Paul Slovic’s work (e.g. Slovic 1992, 1999). The frustration here partly stems from Roeser’s partial agreement with Slovic. On the one hand, Roeser thinks Slovic gets it altogether right when he notes that ordinary people tend to assess risks in ways that take into account a much wider range of considerations than technical experts do in their risk assessments. Slovic also gets things right when he explains this with reference to the role that emotions play in ordinary people’s judgments about risks. But on the other hand, Roeser finds it disappointing when Slovic assumes that the values that ordinary people appeal to in their risk evaluations are subjective or socially constructed. Slovic should not simply assume this, Roeser thinks. Values can be objective, and emotions can be rational (Roeser 2018, 37–38).

In response to views like the ones just surveyed above, Roeser’s overall mission is to present an alternative understanding of values, emotions, and the rationality of laypeople. In presenting this view, one of the key things Roeser aims to do is to vindicate common sense or, in other words, ordinary people’s views about what matters in the ethics of technological risks. But what exactly do emotions have to do with all of this? Why does Roeser think that we should listen to our emotions when we make ethical assessments of technological risks?

## Roeser’s Defense of Risk Assessment Based on Emotions

As I read her, what Roeser most importantly wants us to accept when it comes to the assessment of technological risks are the following two claims:

1. There is a broad range of values (e.g. well-being, fairness, autonomy, equality, and so on) that bear on which technological risks are acceptable and which are not;
2. When we assess specific technological risks, we should consult our emotions.

Much of Roeser’s book is about painting an overall picture that helps to vindicate those two claims. Some of the book does this by discussing the nature of emotions. Some of the book does this by offering a meta-ethical theory that makes room for both a plurality of values and for an understanding of our emotions as rational. However, I believe that much of Roeser’s meta-ethical machinery is not needed in a defense of (1) and (2). Moreover, much of Roeser’s meta-ethical machinery is highly controversial. Luckily, what I will call the first part of Roeser’s defense of consulting our emotions is a very simple argument that can offer a powerful defense of (1) and (2) without the need for any controversial meta-ethical theories.

Let’s start, then, with the simple and powerful argument. As I understand it, that argument can be restated as follows:

Premise 1: One of the key things our emotions do is to make clear to us what our values are: they “point” us towards our values.

Premise 2: In ethical reasoning, we should make use of the full range of our values.

Conclusion: In ethical reasoning—about technological risks or any other topic—we should consult our emotions.

A few comments about this argument and its premises: The first premise is one that Roeser repeats many times throughout the book (Roeser 2018, e.g. 2–3; 6–7; 20; 23; 35; 112). In my assessment, this is an eminently plausible premise. To be sure, if we only focus on very intense emotions experienced on single occasions, then these can sometimes rightly be seen as clouding us from some of our values. In the heat of the moment, a person might be so angry or afraid that they forget about the importance of tolerance or honesty. But over time, our emotions help us to make clear to us what things are most important to us in life. After all, to value something is, inter alia, to care about it in a robust and emotionally involved way (Tiberius 2008). Accordingly, I take it that Roeser is right in repeating variations of premise one several times in her book.

What about premise two? It would be very odd to reject this premise. If you did, you might say things like, “in ethical reasoning, we should set aside some of our values and only focus on some small sub-set of our values. For example, we could choose to focus on the sub-set of our values that are related to efficiency and profit-maximizing.” This, however, would not sound as much as a serious ethical argument as it would sound like somebody trapped in a business- or managerial mindset. Or you might say something like “actually, even though we might have a range of values, we should dismiss some of our values and only focus on the values that technical experts claim that we should focus on. In fact, we can outsource our ethical reasoning to them.” This would not so much be an ethical argument as a refusal to responsibly participate in ethical arguments and doing so in a way that stays true to one’s values. I take it that we should accept the second premise. Rejecting it would amount to not doing full justice to the richness of what ethical argumentation can amount to.

One more note about premise two: I do not wish to claim that all ethical values should always be given the same weight or priority in all types of situations or within all different domains of life. That is not the point of premise two as I understand it. For instance, if faced with an extreme situation—such as a natural disaster—people can sensibly set certain otherwise important values (such as sustainability) aside momentarily and for the moment primarily focus on, say, saving lives and values like solidarity. Or if we compare different domains (e.g. traffic versus education), it can make sense to view some values as being more important in relation to some of these domains and other values as being more important in relation to other domains. But that our ethical reasoning ought ideally to be sensitive to differences across situations and domains does not mean that an ethically responsible person or community should seek to narrow down the range of ethical values they take into account nor that they should outsource their ethical reasoning to supposed experts only focused on some specific type of reasoning. Rather, an ethically responsible person or community should (among other things) try to do two different things that can admittedly be hard to do in practice: (1) be sensitive to whether the situation or domain at hand calls for the prioritization of some values over others, and at the same time (2) be mindful of how prioritizing those values in the given situations or domains could be made to harmonize with respecting or honoring the full range of ethically relevant values as we move between different situations and domains. In other words, recognizing that some values can sometimes become more salient or more important in some contexts does not mean that other values suddenly become irrelevant.^1^ As I understand premise two, then, it is not a call to always treat all values equally in all situations, but rather a call to take into account the full range of our ethical values in a nuanced and measured way, which is perfectly compatible with being sensitive to ethically relevant differences among different situations and domains (cf. Dworkin 2013).

About the conclusion the only thing I will note here is that it does not imply that our emotions are the only things we should consult when we engage in ethical reasoning. Nor does Roeser suggest that in her book. Rather, the idea is that our emotions are among the things we should consult when we try to formulate ethical arguments. Other things we can consult include more abstract theories of values, general moral principles, our assessments of probabilities, causal beliefs about the relations among different things and processes, and so on and so forth. The conclusion of Roeser’s argument is not the extreme view that emotions are the only things we should consult, but rather that they are one of the things we should consult in ethical reasoning about risks. Like the two premises, the conclusion of this argument is very reasonable.

But like I said, what I am calling this simple and powerful argument is not the only part of Roeser’s defense of (1) and (2). Her defense of these claims also has a second part. That second part is a certain interpretation of meta-ethical moral realism, which has a special role for emotions in it (Roeser 2018, 85).

In general, moral realism is a family of views postulating the existence of ethical facts or truths that obtain independently of what our actual attitudes or ethical convictions are. Moral realism, then, is not a first-order ethical theory about how to live our lives, as it is typically conceived. It is rather a higher-order theory about the status of our moral convictions and their relations to what are thought to be independent ethical truths or facts. Simply put, moral realists believe that there are moral facts, that we sometimes track those moral facts with our moral convictions, and that when people disagree about moral matters, some might be getting it right, whereas others might be getting it wrong. When people do get things right, it is because they are somehow able to comprehend, realize, intuit, or otherwise make a connection with independent moral facts or truths (Sayre-McCord 2017).

It is in relation to this last-mentioned point—i.e. the issue of how to get in touch with moral facts or truths—that the second part of Roeser’s view about why we should consult our emotions in ethical reasoning comes into play. Roeser holds the view that “through emotions we can directly perceive objective moral aspects of the world” (Roeser 2018, 91). Emotions, in other words, do not just point inwards towards our values, motivations, and thoughts about things. Emotions also point outwards, so to speak. They function as a form of perception of the world. In particular, emotions are perceptions of “moral aspects” of reality (Roeser 2018, 91). On this picture, then, we perceive non-moral aspects of reality with our senses, and moral aspects of reality with our emotions. This type of “affectual intuitionism”, as she calls it, is what I am calling the second part of Roeser’s defense of why we should consult our emotions in ethical reasoning (cf. Roeser 2011). According to this second part, we should consult our emotions because some of them are perceptions of moral aspects of the world. We should consult them because they help us to get in touch with non-subjective and mind-independent moral facts about how to interact with other people or risky technologies.

Is this second part of Roeser’s defense of the claims (1) and (2) as simple and powerful of an argument as the first part of her defense of claims (1) and (2) is? I will now discuss that question by considering Roeser’s form of moral realism in the context of some wider meta-ethical debates and, in particular, other recent forms of moral realism.

## The Controversial Nature of Roeser’s Meta-ethics

The website PhilPapers.org ran a survey with all its users around 10 years ago, to see what philosophical views the website users “accept or lean towards”. The users are primarily academic philosophers, and one of the choices on the survey was among “moral realism”, “moral anti-realism”, and “other” meta-ethical theories. In that survey, 56.4% responded that they accept or lean towards moral realism.^2^ So in terms of sheer numbers, it appears that about half of all Anglophone philosophers prepared to participate in this type of survey accept or lean towards some form of moral realism, whereas the other half accepts or leans towards different forms of moral anti-realism or some unspecified “other” theory. Of course, it is not clear from the survey what kinds of moral realism and moral anti-realism are meant. And if those are the only options to choose from, the categories of moral realism and moral anti-realism—as well as the category of “other” theories—become very broad.

When it comes to those who defend moral realism in their recent published work—and who do not just indicate that they accept or lean towards it in surveys—most authors defend forms of moral realism that are quite different from the variety that Roeser defends. As I read them, most other moral realists would not be happy with the thesis that we are able to perceive moral aspects of reality. That, they would instead argue, is the sort of view critics of moral realism tend to understand by moral realism, not what most moral realists these days themselves believe. Christine Korsgaard, for example, caricatures moral realists as claiming that we can spot moral entities “wafting by” (Korsgaard 1996, 44). Ronald Dworkin, who is a type of moral realist himself, makes fun of the view that we can causally interact with moral properties by saying that this would require there to be some sort of moral particles we could interact with, which we might call “morons” (Dworkin 2013, 43). Similarly, Allan Gibbard—who defends what he calls a form of “quasi-realism”—writes that if anybody “seriously believes” that we can intuit mind-independent moral facts in some perceptual sort of way, he simply wants to “debunk it” (Gibbard 1992, 154).

If the view that we can “perceive moral aspects of reality” is so controversial and many philosophers do not even take it seriously, what do other moral realists believe? Three influential moral realists who have recently defended more slimmed-down versions of moral realism are Nagel (2012), Scanlon (2016), and Parfit (2017). They all agree with the non-subjectivism of Roeser, and argue that moral facts or truths are not constituted by facts about our attitudes or emotions. But at the same time, they also deny that moral realism needs to posit any moral aspects of reality. Moral truths, as Parfit puts things, have no “ontologically weighty” implications (Parfit 2017, 183). Unless we use “reality” in some very general sense, reality contains no properties or entities beyond what can be described by the natural and social sciences. Moral properties and truths do not exist anywhere in space or time, and cannot be perceived. Instead, we come into contact with moral truths, on this view, by thinking hard about how to live our lives and by coming to have justified and true beliefs that are well-supported by good moral arguments (Dworkin 2013). We accept some propositions about values or moral principles based solely on the inherent plausibility of those propositions—Parfit’s favorite example is that agony is bad—but this does not amount to perceiving any distinctive aspects of reality (Parfit 2017).

According to writers like Dworkin, Nagel, Scanlon, and Parfit, this is the sort of moral realism that we need to adopt if we want to formulate a version of realism that can have broad appeal among philosophers so that we can hope to potentially convert skeptics to the realist’s cause. On their view, the sort of view that Roeser puts forward is far too controversial and implausible for either realists like them or skeptics about moral realism to be willing to accept it.

In pointing this out, I do not mean to suggest that Roeser is wrong in holding her view and that the versions of moral realism defended by Parfit and others are more likely to be correct. For all I am assuming in my argument here, Roeser might be right, and most other contemporary moral realists might be wrong. My point here is rather that Roeser’s view is very controversial. It is a view that most philosophers—including those who accept or lean towards moral realism—do not accept. And the further point I wish to make is that if our defense of consulting our emotions in ethical reasoning about risk rests on the controversial type of moral realism Roeser advocates, then our chances of finding wide acceptance among philosophers are slim. That would be bad publicity for the view that we should consult our emotions. It would put that view on very shaky ground. It would do so even if most moral philosophers are wrong and Roeser is right.

Like I said above, however, I do not think that Roeser needs her meta-ethical realism in order to defend the view that we should consult our emotions when we engage in ethical reasoning about technological risks. Roeser’s simple and powerful argument discussed in the foregoing section by itself helps to establish this. It does so without the need for the additional controversial meta-ethical views that Roeser holds but most philosophers, including most moral realists, do not agree with. For this reason, we can consider the second part of Roeser’s defense of the theses (1) and (2) as one possible meta-ethical story about how values and emotions should be interpreted, but as an optional story we do not necessarily need to subscribe to in order to agree with Roeser that there is reason to consult our emotions in ethical reasoning.

## A Possible Reply from Roeser

At one point in her book, Roeser offers an argument that might be seen as a response to what I just said above about her meta-ethical realism being an optional part of her view. Roeser discusses subjectivism and social constructivism about ethics, and she argues as follows. If we accept any other view than moral realism, values become arbitrary, and there is no longer any point to taking ethical discussions seriously. We “might just as well throw dice or appoint a dictator to determine what to do” unless there are true and correct answers to how we should live our lives and interact with new technologies (Roeser 2018, 38). Serious moral reasoning, Roeser thinks, assumes that there are some true moral beliefs and that there are solid moral facts that those true moral beliefs are tracking. If it is correct that serious moral reasoning and sincere moral debates indispensably require us to presuppose that we can either get things right or wrong and that there, therefore, are moral facts, perhaps this could also be seen as argument for postulating perceivable “moral aspects of reality”, like Roeser does. Would this be a good reply to the reasoning above?

My assessment is that this would not be a strong reason for supposing that we have intuitive abilities to perceive moral properties. That serious disagreements presuppose that one party could be right and the other wrong does not by itself have to have any ontological implications about what types of properties or entities there are in the world. All that it implies is that there is a distinctive type of reasoning we can use to satisfy ourselves that one side of some issue has a stronger argument to back up their case with than the other side does (Scanlon 2016).

We do not settle moral disagreements by ontological investigations into what exists and what does not exist in the moral domain. Rather, we try to settle moral disagreements by formulating moral arguments that we present each other with (Dworkin 2013). An argument to the effect that “but I am perceiving that it is wrong to do such-and-such!” is not going to count as a strong argument that others have reason to accept. Rather, ethical arguments put forward values or ethical principles and relate these to features or aspects of the situation or the problem at hand. Saying that one party to a disagreement has less advanced intuitive powers or less well-developed moral perception is not a good and respectful way to try to settle any moral dispute. Nor do I think that Roeser would herself recommend the use of such arguments in ethical deliberations, given her very sensible account of proper moral deliberation that she puts forward in the last part of her book (Roeser 2018, 141–168).

I conclude, then, that even if Roeser is right that serious moral arguments presuppose that some points of view are better or more correct than others, this has no ontological implications in terms of what exists or does not exist in the universe. It only implies that we have standards for evaluating arguments and methods for formulating moral arguments that we can expect others to take seriously whether they ultimately accept them or not. This might push us in the direction of the sort of light-weight moral realism that Dworkin, Nagel, Scanlon, Parfit, and others are defending. But it does not need to push us in the direction of the more extravagant form of moral realism that Roeser refreshingly defends.

## Footnotes

1 In his 2006 book about scandals in the business world and why they happen, Kenneth Goodpaster argues that one of the common features of many scandals is that people become fixated on domain-specific goals and thereby ignore the full range of values that they otherwise typically adhere to in their day-to-day life. The key to avoiding scandals, Goodpaster argues, is in part to try to pursue domain-specific values and goals in a way that is sensitive to and that takes into account the full range of human values (Goodpaster 2006).

2 https://philpapers.org/surveys/results.pl this survey had a total of 931 participants.
